# The Neural Correlates of the Clock-Drawing Test in Healthy Aging

**DOI:** 10.3389/fnhum.2019.00025

**Published:** 2019-02-05

**Authors:** Natasha A. Talwar, Nathan W. Churchill, Megan A. Hird, Iryna Pshonyak, Fred Tam, Corinne E. Fischer, Simon J. Graham, Tom A. Schweizer

**Affiliations:** ^1^Keenan Research Centre for Biomedical Science, St. Michael’s Hospital, Toronto, ON, Canada; ^2^Institute of Medical Sciences, University of Toronto, Toronto, ON, Canada; ^3^Physical Sciences Platform, Sunnybrook Research Institute, Toronto, ON, Canada; ^4^Department of Psychiatry, Faculty of Medicine, University of Toronto, Toronto, ON, Canada; ^5^Department of Medical Biophysics, Faculty of Medicine, University of Toronto, Toronto, ON, Canada; ^6^Division of Neurosurgery, St. Michael’s Hospital, Toronto, ON, Canada; ^7^Institute of Biomaterials and Biomedical Engineering, University of Toronto, Toronto, ON, Canada

**Keywords:** clock-drawing test, healthy aging, functional MRI, cognitive assessment, brain mapping

## Abstract

**Importance:** The clock-drawing test (CDT) is an important neurocognitive assessment tool, widely used as a screening test for dementia. Behavioral performance on the test has been studied extensively, but there is scant literature on the underlying neural correlates.

**Purpose:** To administer the CDT naturalistically to a healthy older aging population in an MRI environment, and characterize the brain activity associated with test completion.

**Main Outcome and Measure:** Blood-oxygen-level dependent (BOLD) functional MRI was conducted as participants completed the CDT using novel tablet technology. Brain activity during CDT performance was contrasted to rest periods of visual fixation. Performance on the CDT was evaluated using a standardized scoring system (Rouleau score) and time to test completion. To assess convergent validity, performance during fMRI was compared to performance on a standard paper version of the task, administered in a psychometric testing room.

**Results:** Study findings are reported for 33 cognitively healthy older participants aged 52–85. Activation was observed in the bilateral frontal, occipital and parietal lobes as well as the supplementary motor area and precentral gyri. Increased age was significantly correlated with Rouleau scores on the clock number drawing (R2) component (rho = -0.55, *p* < 0.001); the clock hand drawing (R3) component (rho = -0.50, *p* < 0.005); and the total clock (rho = -0.62, *p* < 0.001). Increased age was also associated with decreased activity in the bilateral parietal and occipital lobes as well as the right temporal lobe and right motor areas.

**Conclusion and Relevance:** This imaging study characterizes the brain activity underlying performance of the CDT in a healthy older aging population using the most naturalistic version of the task to date. The results suggest that the functions of the occipital and parietal lobe are significantly altered by the normal aging process, which may lead to performance decrements.

## Introduction

The clock-drawing test (CDT) is important for evaluating mental status in both neurological and psychiatric populations (Freedman et al., 1994). It is applied widely for Alzheimer’s disease (AD) and related dementias, along with many other neurocognitive disorders such as Huntington’s disease and Parkinson’s disease (Rouleau et al., 1992; Shulman, 2000; Yamamoto et al., 2004; Pinto and Peters, 2009). Although simple and easy to administer, performance of the CDT depends on successfully engaging multiple cognitive functions including visuospatial processing, executive function, semantic memory, and planning (Rouleau et al., 1992; Freedman et al., 1994; Royall et al., 1998, 1999; Shulman, 2000; Paula et al., 2013). Due to the multi-domain nature of the task, it has proven to be an effective screening tool for AD and dementia with a sensitivity range of 76–87% and specificity range of 78–87% depending on the scoring method employed (Watson et al., 1993; Brodaty and Moore, 1997; Kirby et al., 2001; Nishiwaki et al., 2004; Velayudhan et al., 2018).

Many studies have evaluated the efficacy of the CDT as a clinical assessment (Kozora and Cullum, 1994; Shulman, 2000; Yamamoto et al., 2004; Pinto and Peters, 2009; Ehreke et al., 2010), but very little has been reported on the neuroanatomical underpinnings of successful CDT performance in healthy individuals and patients. Given the widespread use of the CDT as a neuropsychological assessment tool, it is essential to determine which brain regions are involved in this test in a healthy older population in order to identify brain areas in which pathological aging may lead to impaired CDT performance. A critical area of healthy aging is the transition from older middle age to >80 years of age, as this is when the prevalence of dementia substantially increases (Ott et al., 1998; van der Flier and Scheltens, 2005). Research has shown a relationship between CDT scores and age, with performance declining in the elderly population (Kozora and Cullum, 1994; Paganini-Hill et al., 2001; Hubbard et al., 2008).

Early neuroimaging work has been done to investigate the neural correlates of the CDT (Ueda et al., 2002; Ino et al., 2003; Lee et al., 2008; Thomann et al., 2008; Tranel et al., 2008; Matsuoka et al., 2010, 2013). Researchers have used single photo emission computed tomography (SPECT) to correlate resting regional cerebral blood flow with CDT score and found significant relationships with the bilateral parietal and posterior temporal lobes, right middle frontal gyrus and right occipital lobe (Ueda et al., 2002; Matsuoka et al., 2013). Other studies have implicated the bilateral temporal lobes, right parietal lobe, and the frontal cortex using magnetic resonance imaging (MRI) to measure the correlation between gray matter density and CDT score (Cahn-Weiner et al., 1999; Thomann et al., 2008; Matsuoka et al., 2010).

Beyond these methods, functional MRI (fMRI) provides a tool capable of identifying networks of activity throughout the whole brain during CDT performance, based on the blood oxygenation level-dependent (BOLD) signal contrast mechanism (Ogawa et al., 1990). Despite the heavy usage of fMRI in neuroscience, there are special ergonomic and engineering challenges associated with realistically implementing the CDT in such imaging studies, because the test requires individuals to communicate in writing while lying in the magnet bore (Tam et al., 2012). Only one previous task-based fMRI study has reported brain activity during a modified version of the CDT (Ino et al., 2003), in which participants traced clock hands using their finger on a plastic board after receiving auditory instructions. The participants did not complete the entire clock drawing task, used their finger to draw and had no visual feedback of their actions- all of which may contribute to substantially different task demands and underlying brain regions compared to the CDT, limiting interpretation of the fMRI results. Shoyama et al. (2011) used multichannel near-infrared spectroscopy (NIRS) to overcome the limitations of an MRI environment and measure hemoglobin concentration changes during a traditional version of the CDT (Shoyama et al., 2011). However, the study only measured hemoglobin changes in the bilateral prefrontal and superior temporal cortical surface regions, providing limited information about the whole-brain response during CDT performance.

The present study extends prior research by employing novel fMRI-compatible touch-sensitive tablet technology with real-time visual feedback of hand and stylus position (Karimpoor et al., 2015), to investigate performance and neural correlates associated with the CDT in a much more realistic testing environment. This initial work aimed to examine the underlying neural correlates of the CDT in a healthy older aging population, hypothesizing that (1) the test engages extensive areas of the brain including the bilateral frontal and parietal lobes; and that (2) decreases in task-related activity in key frontal and parietal areas are associated with age-related reductions in task performance.

## Materials and Methods

### Participants

Thirty-seven (*n* = 37) cognitively healthy older participants aged 52–85 were recruited into the study from the local community. All participants had to meet MRI screening criteria, which ensured they had no metal in their body or any other safety hazards that would pose a threat during the MRI scanning procedure. Participants were excluded if they had a history of any of the following: stroke, traumatic brain injury, brain tumor, seizure, any neurological condition (e.g., Parkinson’s disease, Alzheimer’s disease, multiple sclerosis), any psychiatric condition (e.g., bipolar disorder, schizophrenia), a gross movement disorder or impairment or substance abuse. No participants had any visual abnormalities that were not corrected with lenses or significant hearing loss. All participants were right handed based on the Edinburgh Handedness Inventory (Oldfield, 1971) and were fluent in English. All provided written informed consent to participate in the study, which was approved by the Research Ethics Board at St. Michael’s Hospital, Toronto, Canada. Four participants were excluded from the analysis: one had an outlying Montreal Cognitive Assessment (MoCA) score (see section Analysis of Behavioral Measures); one was unable to follow instructions; one had non-reproducible brain activity; and one had significantly elevated head motion (see section fMRI Preprocessing and Analysis).

### Psychometric Testing

The cognitive abilities of the participants were assessed prior to the MRI session using the MoCA (Nasreddine et al., 2005), which is a commonly used cognitive assessment battery, and the CDT. This work was conducted in the context of a larger fMRI study that included other neurocognitive tests (i.e., letter cancelation test, trail-making test, mazes), which were administered at this time to provide validation for the tablet versions of these tests. All tests were led by an experienced test administrator (MH, NT). For the CDT, participants were given a blank piece of paper and a pen, and instructed to “draw a large circle, put all the numbers in to make it look like the face of a clock, draw the hands of the clock to show 10 min after 11 and to stop when completed.” The time to complete the entire CDT was recorded with no maximum time allotted. After the fMRI session, the participants completed the post-experimental tablet questionnaire, which provided self-reported ratings on task performance and comfort during the MRI session, and the Edinburgh Handedness Inventory.

### Magnetic Resonance Imaging

Participants were imaged at St. Michael’s Hospital using a 3.0 Tesla MRI system with the standard 20-channel head coil (Magnetom Skyra, Siemens Healthineers, Erlangen, Germany). Structural images were acquired using a three-dimensional T1-weighted Magnetization Prepared Rapid Acquisition Gradient Echo protocol (MPRAGE: inversion time (T1)/echo time (TE)/repetition time (TR) = 1090/3.55/2300 ms, flip angle (FA) = 80°, bandwidth (BW) = 200 Hz/px, sagittal orientation with field of view (FOV) = 240 mm by 240 mm by 173 mm, 256 by 256 by 192 acquisition matrix, isotropic voxel dimension = 0.9 mm thickness). The fMRI data were acquired during CDT performance using two-dimensional multi-slice T2^∗^-weighted echo planar imaging (EPI: TE/TR = 30/2000 ms, FA = 70°, BW = 2298 Hz/px, oblique-axial, slices interleaved ascending, with FOV = 200 by 200 mm, 64 by 64 acquisition matrix, 32 slices with 4.0 mm thickness and 0.5 mm gap, voxels = 3.125 mm by 3.125 mm by 4.0 mm).

### fMRI-Compatible Tablet Technology

**FIGURE 1:**
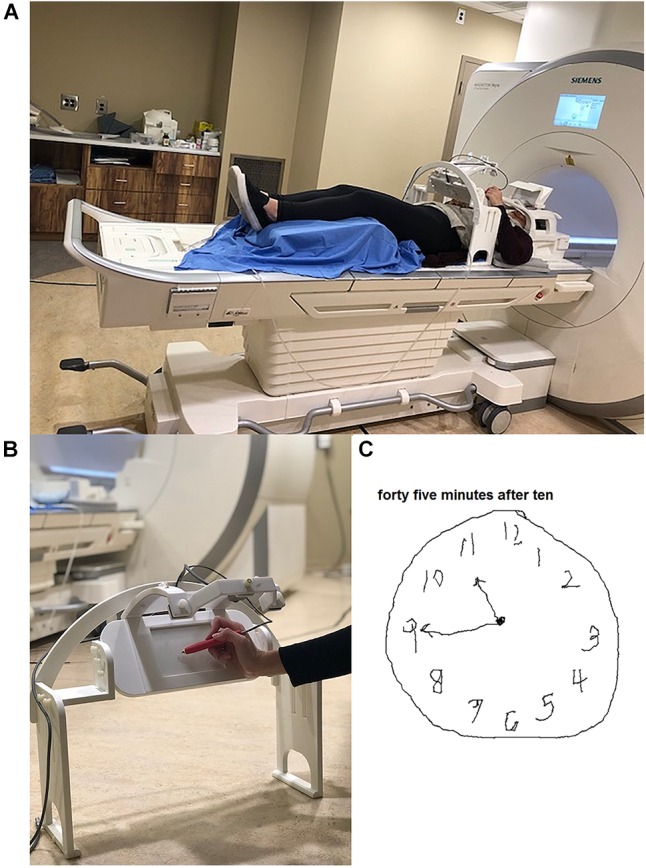
Tablet technology used in the study. **(A)** Set-up on the patient table prior to position in the magnet bore, showing tablet and mount located at the waist of the participant, and placement in the standard head coil including the angled mirror. An individual from our lab modeled the tablet set-up and gave consent for publication of this image. **(B)** Close-up of the mount, tablet and video camera placement during tablet interaction. **(C)** Example of a completed clock drawing test by a participant using the tablet in the scanner.

The MRI-compatible tablet has a touch screen and stylus to provide a realistic approximation of pen and paper conditions, as well as an augmented reality system providing visual feedback of hand and stylus position for fine motor control (Karimpoor et al., 2015). During imaging, participants lay supine with an adjustable mount over their waist to hold the tablet and camera in place, which enabled them to make precise tablet interactions with their arm supported. A mirror was angled at the top of the head coil enabling participants to view task instructions and their tablet interactions on a display screen that was illuminated by an MR-compatible projector (Avotec, Stuart, FL, United States). A picture of the set-up is shown in Figure 1. Participants were provided with pads underneath their elbows for comfort when performing tablet interactions. fMRI-compatible prescription glasses (MediGlasses for fMRI, Cambridge Research Systems, Kent, United Kingdom) were provided for participants as necessary.

### fMRI of the CDT

The tablet-based CDT was presented to participants using commercially available software for behavioral testing (E-Prime Version 2.0, Psychology Software Tools, Inc., Sharpsburg, PA) run on a personal computer. The CDT was designed by adapting the Ministry of Transport Ontario (MTO) version of the task (Ministry of Transportation Ontario, 2018). Before beginning, the participants were briefed on the experimental set-up and performed a training session to familiarize them with the tablet. Participants also completed a practice session in the magnet bore where they performed simple tasks (write your name, trace a flower, etc.) and were qualitatively assessed to ensure they were able to manipulate the stylus.

**FIGURE 2:**
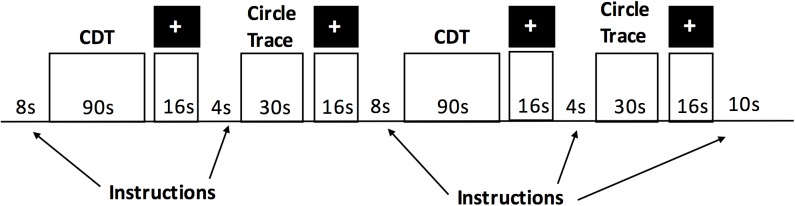
The experimental protocol for a “run” of the tablet-based CDT session during fMRI.

The tablet-based cognitive testing involved a battery of different tasks, which were presented in a randomized order. The CDT was one of the tasks in this battery. The tablet-based CDT protocol consisted of two “runs” (total time duration = 14 min). There were approximately 12 min of other cognitive tasks interleaved between the two CDT runs. The task design for each CDT run is depicted in Figure 2 and consisted of three condition blocks: the clock-drawing test (CDT), a circle tracing condition and a fixation condition. The order of the three conditions (CDT, circle tracing and fixation) was fixed in order to provide periods of rest (visual fixation) between each of the CDT and circle tracing blocks. For the CDT, participants were instructed by a text display to “draw a large circle. Put all the numbers in to make it look like the face of a clock. Draw in the hands of the clock to set the time to the time specified. Stop when completed.” Participants were then given 90s to comply with the instructions. Over the two runs, participants performed five trials of the CDT while setting the time to 3 o’clock, 20 min to 4, 10 min after 11, 45 min after 10, and 5 min after 6. Multiple clock times were chosen to minimize practice effects between blocks, with the first time condition (3 o’clock) selected as a simple instruction to help participants adjust to the task. The order of the five CDT time conditions was held fixed. For the circle tracing condition, participants were instructed to continuously trace a circle. They were presented with a pre-drawn circle on the display and continuously traced the outline of the circle for a total of 30 s, as quickly as possible while maintaining accuracy. During the fixation task, participants were asked to fixate their attention on a black cross in the middle of the display for 16 s.

### Analysis of Behavioral Measures

Two different metrics were used to evaluate in-scanner performance on the CDT: score and time to complete. The CDT results were scored using the method outlined by Rouleau et al. (1992), which is a semi-qualitative scoring method that consists of three components: R1, used to assess the integrity of the clock face, maximum score = 2; R2, used to assess the presence, sequencing and spatial arrangement of the numbers within the clock, maximum score = 4; and R3, used to assess the presence and placement of the clock hands, maximum score = 4. Higher scores indicate better performance on the task, for example a R1 score of 0 would indicate an inappropriate depiction of a clock face, whereas a score of 2 would indicate a present clock face without gross distortion. This scoring method allows measurement of performance on each individual component of the task, providing more detail on what types of errors are being made. The Rouleau method is commonly used in CDT research, particularly in groups with older populations and dementia because it measures error types (Chiu et al., 2008; Ehreke et al., 2010; Siciliano et al., 2016; Spenciere et al., 2017). The total score (sum of R1, R2, and R3) was also calculated. Each clock was scored separately by two different individuals to ensure reliability of the scoring method. In the rare event of discrepancy in the score between the two raters (16% of the time), the average of the two scores was used for analysis. In addition, Cohen’s Kappa inter-reliability was used to quantify the level of agreement between the two individuals who scored the CDT for each score (R1, R2, R3, and total). The time to complete metric for the CDT consists of the drawing durations of each clock component as well as the completion time for the whole clock. The behavioral measures, including CDT scores and time to complete the clock components and total, were averaged for each participant using data from both CDT runs during fMRI. Therefore, the average performance for each participant included data from all five tablet-based clock conditions (3 o’clock, 20 min to 4, 10 min after 11, 45 min after 10, and 5 min after 6). Average performance was correlated with age using a Spearman’s rank correlation. Outliers were determined using the MoCA score to prevent significant cognitive impairment or enhancement from skewing the neurocognitive results for the group. Outliers were classified as MoCA scores that were 2.5 standard deviations outside of the group mean MoCA score. The upper limit exceeded the maximum achievable score on the MoCA. One subject had an outlying MoCA score that was below the lower limit (15, p ∼ 0.01, Normal distribution, 2-tailed). The analogous scores were generated for performance of the standard pen-and-paper CDT. The results for each score (R1, R2, R3, and total) were then compared to the tablet-based CDT results using a Spearman’s rank correlation to assess convergent validity. Only the performance of the third tablet-based CDT trial was used in the comparison, so that the time instructions were matched (i.e., “ten past eleven”).

### fMRI Preprocessing and Analysis

The fMRI data and structural scans were manually inspected for any visual abnormalities. With none found, data preprocessing and analysis were then performed using a hybrid pipeline, which included tools from the Analysis of Functional Neuroimages (AFNI)^1^ package (Cox, 1996), the FMRIB Software Library (FSL)^2^ package (Smith et al., 2004) and algorithms custom-written in the laboratory. The preprocessing pipeline incorporated slice-timing correction (AFNI *3dTshift*), rigid-body motion correction (AFNI *3dvolreg*), spatial smoothing (AFNI *3dmerge*), removal of outlier scan volumes via SPIKECOR (Campbell et al., 2013), and regression of motion parameters and linear-quadratic trends as nuisance covariates. To control for physiological noise, a) PHYCAA+ (Churchill and Strother, 2013) was used to perform data-driven down-weighting of regions other than gray matter, without the need to acquire cardiac or respiratory waveforms during imaging; and b) seed-based regression of white matter and CSF was performed using regions of interest in the left and right corona radiata and the left and right lateral ventricles. The resulting data were transformed into a common neuroanatomical template space as follows: the FSL *flirt* algorithm was used to calculate the rigid-body transform of the mean fMRI volume to the T1-weighted anatomical image, and the affine warp of the T1 anatomical image to the MNI152 (Montreal Neurological Institute) template (Mazziotta et al., 1995) for each participant. The net transformation matrices were applied to the fMRI data, which were resampled at an isotropic spatial resolution of 3 mm. Due to the variability in brain size among participants >80 years old, the anatomical transformation was improved by manual inspection and manual segmentation of the brains, if required.

Analysis of the preprocessed imaging data at the individual subject level (first level) was completed by fitting the task conditions (CDT, circle tracing and fixation) in an ordinary least squares general linear model (GLM), to measure the contrast of CDT performance vs. fixation. Activation during all tasks (CDT, circle tracing and fixation) in both runs was used in the analysis, therefore activity during all five CDT conditions (3 o’clock, 20 min to 4, 10 min after 11, 45 min after 10, and 5 min after 6) was considered. The circle tracing task involves fine motor and visuospatial abilities, making it a challenging task that is not an appropriate control for clock-drawing, therefore the CDT vs. circle tracing contrast was not analyzed in this study. The analysis was done in the NPAIRS (Strother et al., 2002) analysis framework, with regression coefficient maps of runs 1 and 2 calculated separately, before combining to obtain a reproducible, Z-scored map of activation, along with a measure of between-run reproducibility (i.e., Pearson correlation of the pairwise voxel values). To ensure that results are not influenced by subjects with excess head motion or poor BOLD signal in this older cohort, two tests were performed on fMRI data. The first test used the motion parameters derived from motion correction, measuring median absolute displacement on each of the 6 rigid-body motion axes for every subject. We then fit a gamma distribution to each parameter and identified any subjects with significantly elevated displacement at *p <* 0.05 (Bonferroni adjusted). One subject had outlying head motion on the yaw axis (0.75°; group median [interquartile range (IQR) = 0.19° (0.09°); gamma test, *p* < 0.001]. The second test examined between-run reproducibility values and identified any subjects with reproducibility below zero, indicating that CDT-related brain activity was non-reproducible between runs. One subject had non-reproducible brain activity between runs [Pearson correlation = -0.07; group median (IQR) = 0.50(0.26)].

Non-parametric group-level (second level) analysis was then done by performing 1-sample bootstrap analyses on the z-scored participant maps for the whole group with effect size on the regression coefficients based on bootstrap resampling and adjusting for multiple comparisons using a cluster size threshold (*p* < 0.005, cluster size = 20). Covariate analysis was completed using a GLM that included both age and total score as regression coefficients to determine the effect of each variable on the task-related activation.

## Results

### Participants

Thirty-three participants were included in the final analysis. Demographic and neuropsychological data are summarized in Table 1.

**Table 1:** Demographic and neuropsychological assessment scores of the group.

	Median (IQR)	Quartile 1	Quartile 3
Age	71.0 (15.0)	65.0	80.0
Gender (female), (%)	19.0 (57.6%)		
Years of education	16.0 (3.0)	14.0	17.0
MoCA score	27.0 (2.0)	26.0	28.0
**Paper-Based CDT**			
R1 score	2.0 (0)	2.0	2.0
R2 score	4.0 (0.5)	3.5	4.0
R3 score	4.0 (0.5)	3.5	4.0
Total score	9.5 (1.0)	9.0	10.0
Time to complete (seconds)	30.0 (13.0)	23.5	36.4


*Values reported in median (interquartile range) format unless otherwise stated. N, number of observations; MoCA, Montreal Cognitive Assessment; CDT, clock-drawing test; R1, clock face drawing component; R2, clock number drawing component; R3, clock hand drawing component.*

### Behavioral Results

During fMRI, a statistically significant correlation was found between age and CDT score on all components of the task except R1, the clock-face drawing component (Table 2). There was a significant negative correlation between age and drawing the numbers (R2; rho = -0.549, *p* < 0.001), setting the hands to the correct time (R3; rho = -0.502, *p* = 0.003) and overall CDT score (rho = -0.621, *p* < 0.001). On the paper version of the task, there was a negative correlation between age and CDT score on all components of the task, however, only the correlation between total score and age was statistically significant (rho = -0.35, *p* = 0.04) (Table 2). For both the tablet and paper-based CDT, there was a positive correlation between time to complete the task and age, but without statistical significance (Table 2). The Cohen’s Kappa value for inter-rater reliability between the two individuals who scored the tablet-based CDT was 0.82, which is classified as almost perfect (Cohen, 1960). There was a strong correlation between scores on the paper-based CDT and the tablet-based CDT (rho = 0.884, *p* < 0.001), which suggests good convergent validity between the two versions of the task. Post-experimental questionnaires were completed by 18 participants and showed average self-reporting ratings of good performance on the task with no difference in performance between paper-based CDT and tablet-based CDT. Participants reported comfort with the in-scanner set-up and no adverse physiological symptoms during or after the MRI session.

**Table 2:** Analysis of the effect of age on the performance of the paper-based and tablet-based CDT.

	Median (IQR)	Rho	*p*-value	95% CI	
				Lower	Upper
**Paper-Based CDT**					
R1 Score	2.0 (0)	-0.11	0.55	-0.44	0.24
R2 Score	4.0 (0.5)	-0.18	0.32	-0.49	0.17
R3 Score	4.0 (0.5)	-0.21	0.23	-0.52	0.14
Total Score	9.5 (1.0)	-0.35	0.04	-0.62	-0.01
Total Time (seconds)	30.0 (13.0)	0.33	0.06	-0.02	0.60
**Tablet-Based CDT**					
R1 Score	2.0 (0.1)	0.32	0.07	-0.03	0.59
R2 Score	3.6 (0.8)	-0.55	<0.001	-0.75	-0.25
R3 Score	3.1 (0.7)	-0.50	0.003	-0.72	-0.19
Total Score	8.8 (1.4)	-0.62	<0.001	-0.79	-0.35
R1 Time (seconds)	4.8 (2.1)	0.10	0.58	-0.25	0.43
R2 Time (seconds)	22.3 (6.6)	0.26	0.14	-0.09	0.56
R3 Time (seconds)	10.9 (7.2)	0.34	0.054	-0.01	0.61
Total time (seconds)	38.6 (15.3)	0.33	0.06	-0.01	0.61


*Data for the tablet-based CDT averaged across all 5 trials. Data for the paper-based CDT consists of only one trial. Values reported in median (interquartile range) format. n, number of observations; rho, Spearman’s rank correlation of the CDT performance and age; CI, confidence interval; CDT, clock-drawing test; R1, clock-face drawing component; R2, clock number drawing component; R3, clock hand drawing component.*

### fMRI Results

**FIGURE 3:**
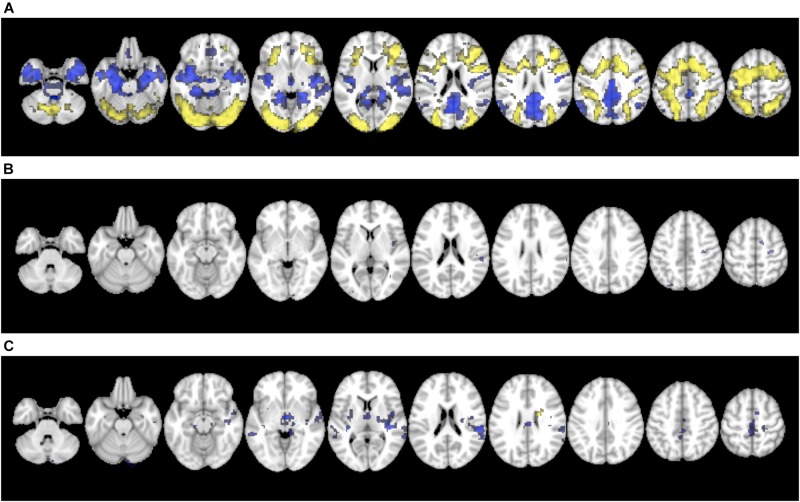
**(A)** Brain activation for the contrast of CDT performance vs. visual fixation. **(B)** Covariance with age, and **(C)** Covariance with performance (total score). Images were displayed in 2 mm × 2 mm × 2 mm resolution for consistency with the anatomical template.

Figure 3A shows that across all participants, an extensive pattern of positive activation (increased activity during CDT performance compared to fixation) was observed in the bilateral frontal cortex, occipital cortex, parietal cortex, inferior temporal cortex, cerebellum, insula, supplementary motor area, middle cingulate gyri, precentral gyri and the left post-central gyrus. Negative activation (decreased activity during CDT performance compared to fixation) was observed in the bilateral insula, temporal cortex, hippocampus, parahippocampal gyri, cerebellum, fusiform gyri, precuneus, cuneus, angular gyri, posterior cingulate gyri and middle cingulate gyri (Figure 3A).

A sparse pattern of brain areas showed reliable effects of age on task-related activation (Figure 3B). All the regions identified showed a negative correlation (decreased task-related activity with increased age) including the right calcarine sulcus, right inferior temporal lobe, left occipital lobe, right insula, right superior temporal lobe, right supramarginal gyrus, right precentral gyrus, left superior parietal lobe and right supplementary motor area.

A similarly, sparse set of brain regions showed effects of total CDT score on task-related activation (Figure 3C). The association was positive (increased task-related activity with increasing total score) in the right caudate nucleus, whereas negative association (decreased task-related activity with increasing total score) was observed in the left cerebellum, right hippocampus, bilateral temporal lobes, bilateral thalamus, right insula, left putamen, right supramarginal gyrus, bilateral middle cingulate gyri, right supplementary motor area and left paracentral lobe. Clusters of activation for Figure 3 are listed in Table 3.

**Table 3:** Clusters of activation during completion of the clock-drawing test.

Cluster number	Cluster size (voxels)	Coordinates of center (x,y,z)			Peak value	Anatomical region
**CDT Performance vs. Fixation (Figure 3A)**						
1	22,727	12,	-39,	9	-0.97	Right posterior cingulate gyrus
2	263	-51,	-63,	3	-5.11	Left middle temporal gyrus
3	196	0,	39,	-15	-4.51	Left gyrus rectus
4	189	51,	-57,	27	-4.79	Right angular gyrus
**Covariance with age (Figure 3B)**						
1	136	27,	-18,	57	-4.91	Right precentral gyrus
2	84	54,	-30,	18	-5.19	Right superior temporal gyrus
3	72	12,	0,	60	-4.65	Right supplementary motor area
4	51	42,	-3,	9	-5.17	Right insula
5	50	15,	-102,	0	-4.59	Right calcarine sulcus
6	38	42,	-66,	0	-4.11	Right middle temporal gyrus
7	38	18,	-48,	66	-4.14	Right superior parietal lobe
8	37	-33,	-93,	12	-4.05	Left middle occipital gyrus
9	24	30,	-66,	-60	-4.16	Right lobe VIII of cerebellum
10	24	-27,	-78,	45	-4.05	Left superior parietal lobe
**Covariance with performance (Figure 3C)**						
1	838	42,	-21,	6	-8.15	Right transverse temporal gyrus
2	219	0,	-33,	48	-4.62	Left middle cingulate gyrus
3	93	-57,	-36,	0	-4.76	Left middle temporal gyrus
4	86	-33,	-18,	6	-4.52	Left insula
5	47	9,	0,	63	-4.76	Right supplementary motor area
6	41	-15,	-27,	-12	-4.40	Left parahippocampal gyrus
7	39	12,	-87,	-27	-4.57	Right crus II of cerebellum
8	35	24,	-30,	63	-3.73	Right post-central gyrus
9	30	18,	-3,	24	4.97	Right caudate nucleus
10	23	-9,	-54,	-33	-3.54	Left lobe IX of cerebellum


*Active clusters of brain areas identified for the clock–drawing test vs. fixation contrast, covariance with age and covariance with performance. Spatial locations of the center of the cluster reported in Montreal Neurological Institute (MNI) coordinates.*

## Discussion

This is the first fMRI study to characterize brain activity during CDT performance for healthy elderly individuals. The study was made possible by the use of novel tablet technology that permitted naturalistic CDT performance during imaging. In contrast with visual fixation, extensive patterns of positive CDT-related activation were observed in the bilateral occipital, parietal, frontal and inferior temporal lobes as well as the bilateral cerebellum, supplementary motor area, precentral gyri and left post-central gyrus. Healthy aging in an older adult population had a significant effect on CDT performance with older adults exhibiting both reduced task-related brain activity and lower behavioral scores.

The positive CDT-related activation results are consistent with our current understanding of the neural correlates of the test (Ino et al., 2003; Thomann et al., 2008; Tranel et al., 2008; Matsuoka et al., 2013), supporting the importance of these brain regions in successful completion of the task. Regarding the visual processing aspects of the CDT, a recent SPECT study reported an association between the hand drawing score (R3) and activation levels in the occipital lobe (Matsuoka et al., 2013). In addition, parietal regions involved in visual and spatial perception, such as the supramarginal gyrus, have been linked to CDT performance in patients with brain lesions (Tranel et al., 2008). The executive function requirements of the test (Royall et al., 1999), critical for controlling and coordinating all elements of the task through to completion, are known to localize in the frontal lobe and more specifically the prefrontal cortex (Roberts et al., 1998; Alvarez and Emory, 2006). The prefrontal cortex was implicated in a NIRS study of the CDT, which reported a significant negative association between task completion time and hemoglobin concentration changes in the prefrontal cortex (Shoyama et al., 2011). The scaffolding theory put forward by Park and Reuter-Lorenz (2009) suggests that with age, the brain develops alternative neural pathways to preserve cognitive functions in older adults despite age-related changes in brain structure (Park and Reuter-Lorenz, 2009). This effect has been localized to the prefrontal cortex, which has shown consistent over-activation in older adults, suggesting that increased activity of this brain area compensates for the decline in structure and function in other brain regions (Cabeza, 2002; Park and Reuter-Lorenz, 2009). The extensive frontal activation during CDT seen in this study is consistent with this theory and implies that recruitment of the frontal lobe allows older adults to complete the task despite changes in neural activity in posterior brain areas. The cerebellum has been associated with higher-level cognitive functions including working memory tasks (Leiner et al., 1993; Desmond et al., 1997), such as the CDT, and was activated in a previous fMRI study that used a less naturalistic implementation of the test (Ino et al., 2003). Finally, the supplementary motor area, precentral gyri and left post-central gyrus were all activated as expected, given their established role in motor performance (Porro et al., 1996; Yousry et al., 1997; Picard and Strick, 2003). Interestingly, the former two areas were activated bilaterally, as often observed for motor tasks with high cognitive demands (Rao et al., 1993; Shibasaki et al., 1993; Cramer et al., 2001). The left lateralization of the post-central gyrus is consistent with the contralateral somatosensory processing component of tablet and stylus interactions made by the test participants, all of whom were right-handed.

In addition to the aforementioned areas, CDT-related activation was observed in the bilateral middle cingulate gyri and insula. The anterior portion of middle cingulate has been implicated in connections with the dorsal prefrontal cortex that are involved in cognition (Stevens et al., 2011; Yu et al., 2011), suggesting that this region may play a role in the executive functioning pathway used during the CDT. The insula and the anterior cingulate cortex are both primary components of the salience network, which functions to recruit and coordinate various brain regions and their behavioral responses to salient stimuli (Menon and Uddin, 2010; White et al., 2010).

This study has provided new information revealing brain areas with reduced activation during the CDT. Negative CDT-related activation was observed in the bilateral insula, hippocampus, parahippocampus, cerebellum, angular gyri, posterior and middle cingulate cortex as well as areas in the temporal and occipital lobes. Many of these regions are involved in the default mode network (Greicius et al., 2003, 2009; Fransson and Marrelec, 2008), which is commonly active during wakeful rest or stimulus-independent thought (Mason et al., 2007) and therefore would be suppressed during a completion of a complex task, such as the CDT. Sub-regions of the insula, middle cingulate gyrus and cerebellum showed contrasting patterns of activation during completion of the CDT. A given brain structure may be responsible for multiple cognitive functions that are localized to distinct sub-regions leading to different levels of activity. For example, the anterior part of the insula is a component of the salience network and was positively activated during task completion (Menon and Uddin, 2010; Uddin et al., 2017).

Activity in the bilateral occipital and parietal lobes was observed to decrease as a function of participant age. Furthermore, a significant negative correlation was found between test performance and age, such that younger adults had better CDT scores (R2, R3, total score) than the older adults. Taken together, these two findings suggest that the reductions in brain activity in the regions identified may be primarily responsible for the CDT performance decrements that occur in old age. Studies investigating changes in cognition due to healthy aging have shown impaired attention, visual scanning and processing, executive function and visuospatial perception in older groups (Grady et al., 1994; Elias et al., 2011; Harada et al., 2013; Miller et al., 2015). All of these elements are required for successful CDT performance. Furthermore, numerous imaging studies have identified age-related changes in the structure and function of the occipital and parietal lobes (Grady et al., 1994; Resnick et al., 2003; Raz et al., 2005; Dennis and Cabeza, 2008; Fjell and Walhovd, 2010).

Increased activation was observed in the right caudate nucleus as a function of participant total CDT score. The caudate nucleus has been implicated in the frontal subcortical circuit for mediating executive function (Mega and Cummings, 1994; Elliott et al., 1997; Funahashi, 2001), and previous anatomical imaging studies have reported correlations between caudate atrophy and impaired CDT performance (Heinik et al., 2000; Samton et al., 2005). Collectively, the present work and prior literature suggest that the caudate nucleus plays an important role in successful test completion. Increased CDT score was also correlated with decreased CDT-related activation in various areas. Previous imaging studies have shown that increased cognitive performance can lead to more efficient brain functioning (Charlot et al., 1992; Rypma et al., 2005; Grabner et al., 2006), suggesting that the negatively correlated areas are less active in more skilled participants due to neural efficiency.

The behavioral performance on the tablet-based CDT showed reasonable convergent validity with the standard version of the test as scored by the Rouleau method (Rouleau et al., 1992). Multiple scoring systems have been devised for the CDT, but the Rouleau method was used here because of its popularity in research studies, especially those involving elderly populations (Yamamoto et al., 2004; Chiu et al., 2008; Ehreke et al., 2009; Siciliano et al., 2016; Spenciere et al., 2017). Our results agree with normative Rouleau scores for an elderly aging group (Cahn and Kaplan, 1997; Turcotte et al., 2018). Future studies could use a more detailed scoring system, but there is currently no strong consensus regarding an optimal scoring system for this task (Richardson and Glass, 2002; Pinto and Peters, 2009; Ehreke et al., 2011; Mainland and Shulman, 2017).

Although this study used novel technology to provide important insight into brain activation patterns of older adults during the CDT, there are a few methodological limitations that are important to note. fMRI is a highly useful tool for studying brain activity, however, the age effect on BOLD signal and low temporal resolution can limit the neuroimaging results of the study. There are physiological effects of aging on the biophysical parameters that are responsible for the BOLD signal contrast mechanism – irrespective of changes in neural activity and this is a limitation commonly seen in fMRI studies with older cohorts (D’Esposito et al., 1999; Garrett et al., 2017). Future studies will be required to corroborate the present initial results, with the inclusion of appropriate physiological imaging measure to provide control data. fMRI provides precise spatial localization of brain activity at the expense of reduced temporal resolution. Other imaging modalities, such as electroencephalogram (EEG), with higher temporal resolution may provide more information about dynamic changes in brain activity during CDT, at the expense of reduced spatial localization. Future studies should ideally combine EEG with fMRI to provide a more comprehensive characterization of CDT-related brain activity. These concerns notwithstanding, the agreement of the present results with the existing literature on the neural correlates of the CDT, including studies involving different imaging modalities, suggests that both the physiological confounds due to aging and the low temporal resolution of fMRI are not having a major impact on the interpretation of the research outcomes.

The five time conditions of the CDT were presented in a fixed order, which is another limitation to the current study. Future studies should randomize the order of the times to counter-balance for practice effects. The cohort of this study was an older aging population between the ages of 52 and 85. The age-related changes that occur along this range have significant importance in both healthy and pathological populations, therefore studying the effect of age on CDT-related brain activity in this group provides valuable insight for both researchers and clinicians. However, it is necessary to conduct further investigations, which explore brain activity during the CDT across a larger age span to more comprehensively characterize the effect of healthy aging.

## Conclusion

This study used novel tablet technology and fMRI to determine the underlying neural correlates of the CDT in a healthy older aging population, providing new insights into the neural mechanisms of the test and their relationship with age and test performance. Across the aging group, positive task-related activity was observed in the bilateral frontal, occipital, parietal and inferior temporal lobes as well as the bilateral cerebellum and key motor areas. There was a significant negative correlation between both performance and task-related activity with age. Decreased activity with older age was seen in the bilateral occipital and parietal lobes, suggesting that function of these areas is affected by normal aging, potentially leading to poorer CDT performance. The CDT is sensitive to cognitive changes due to healthy aging in an older population, which raises questions on its validity as a screening tool for pathological impairment in elderly populations. Further research on this topic is critical because of the widespread use of the CDT as a clinical assessment in older populations. It is necessary to conduct future studies, which compare neural activity during the CDT in a healthy aging and cognitively impaired population to help inform the use of the CDT as a clinical screening tool.

## Ethics Statement

This study was carried out in accordance with the recommendations of the Tri-Council Policy Statement: Ethical Conduct for Research Involving Humans, Canadian Institutes of Health Research, Natural Sciences and Engineering Council of Canada, and Social Sciences and Humanities Research Council of Canada with written informed consent from all subjects. All subjects gave written informed consent in accordance with the Declaration of Helsinki. The protocol was approved by the Research Ethics Board at St. Michael’s Hospital, Toronto, Canada.

## Author Contributions

NT, MH, and IP performed the experiments. NT, MH, IP, CF, and TS were involved in participant recruitment. MH, NC, FT, SG, and TS conceived and designed the experiments. SG, FT, NC, and TS designed the tablet technology. NT, NC, TS, and SG analyzed the data. NT, NC, MH, IP, FT, CF, SG, and TS wrote the manuscript. The authors have not published or submitted any previous papers based on this study and declare no conflicts of interest.

## Conflict of Interest Statement

The authors declare that the research was conducted in the absence of any commercial or financial relationships that could be construed as a potential conflict of interest.

## Footnotes

1 https://afni.nimh.nih.gov

2 https://www.fmrib.ox.ac.uk/fsl

## Abbreviations

CDT: clock-drawing test
MoCA: Montreal Cognitive Assessment
R1: clock face drawing component
R2: clock number drawing component
R3: clock hand drawing component

## References

[B1] Alvarez J. A. Emory E. (2006). Executive function and the frontal lobes: a meta-analytic review. *Neuropsychol. Rev.* 16 17–42. 10.1007/s11065-006-9002-x 16794878

[B2] Brodaty H. Moore C. M. (1997). The clock drawing test for dementia of the alzheimer’s type: a comparison of three scoring methods in a memory disorders clinic. *Int. J. Geriatr. Psychiatry* 12 619–627. 10.1002/(SICI)1099-1166(199706)12:6<619::AID-GPS554>3.0.CO;2-H 9215942

[B3] Cabeza R. (2002). Hemispheric asymmetry reduction in older adults: the HAROLD model. *Psychol. Aging* 17 85–100. 10.1037/0882-7974.17.1.85 11931290

[B4] Cahn D. A. Kaplan E. (1997). Clock drawing in the oldest old. *Clin. Neuropsychol.* 11 96–100. 10.1080/13854049708407036

[B5] Cahn-Weiner D. A. Sullivan E. V. Shear P. K. Fama R. Lim K. O. Yesavage J. A. (1999). Brain structural correlates of clock drawing performance in Alzheimer’s disease. *J. Int. Psychol. Soc.* 5 502–509. 10.1017/S1355617799566034 10561930

[B6] Campbell K. L. Grigg O. Saverino C. Churchill N. Grady C. L. (2013). Age differences in the intrinsic functional connectivity of default network subsystems. *Front. Aging Neurosci.* 5:73. 10.3389/fnagi.2013.00073 24294203 PMC3827623

[B7] Charlot V. Tzourio N. Zilbovicius M. Mazoyer B. Denis M. (1992). Different mental imagery abilities result in different regional cerebral blood flow activation patterns during cognitive tasks. *Neuropsychologia* 30 565–580. 10.1016/0028-3932(92)90059-U 1641120

[B8] Chiu Y. C. Li C. L. Lin K. N. Chiu Y. F. Liu H. C. (2008). Sensitivity and specificity of the clock drawing test, incorporating Rouleau scoring system, as a screening instrument for questionable and mild dementia: scale development. *Int. J. Nurs. Stud.* 45 75–84. 10.1016/j.ijnurstu.2006.09.005 17123533

[B9] Churchill N. W. Strother S. C. (2013). PHYCAA+: an optimized, adaptive procedure for measuring and controlling physiological noise in BOLD fMRI. *Neuroimage* 82 306–325. 10.1016/j.neuroimage.2013.05.102 23727534

[B10] Cohen J. (1960). A coefficient of agreement for nominal scales. *Educ. Psychol. Meas.* 20 37–46. 10.1177/001316446002000104

[B11] Cox R. W. (1996). AFNI: software for analysis and visualization of functional magnetic resonance neuroimages. *Comput. Biomed. Res.* 29 162–173. 10.1006/cbmr.1996.0014 8812068

[B12] Cramer S. C. Nelles G. Schaechter J. D. Kaplan J. D. Finklestein S. P. Rosen B. R. (2001). A functional MRI study of three motor tasks in the evaluation of stroke recovery. *Neurorehabil. Neural Repair.* 15 1–8. 10.1177/154596830101500101 11527274

[B13] Dennis N. A. Cabeza R. (2008). “Neuroimaging of healthy cognitive aging,” in *The Handbook of Aging and Cognition*, eds Craik F. I. M. Salthouse T. A. (New York, NY: Psychology Press), 1–54.

[B14] Desmond J. E. Gabrieli J. D. Wagner A. D. Ginier B. L. Glover G. H. (1997). Lobular patterns of cerebellar activation in verbal working-memory and finger-tapping tasks as revealed by functional MRI. *J. Neurosci.* 17 9675–9685. 10.1523/JNEUROSCI.17-24-09675.1997 9391022 PMC6573411

[B15] D’Esposito M. Zarahn E. Arguirre G. K. Rypma B. (1999). The effect of normal ageing on the coupling of neural activity to the BOLD hemodynamic response. *Neuroimage* 10 6–14. 10.1006/nimg.1999.0444 10385577

[B16] Ehreke L. Luck T. Luppa M. König H. H. Villringer A. Riedel-Heller S. G. (2011). Clock drawing test – Screening utility for mild cognitive impairment according to different scoring systems: results of the Leipzig Longitudinal Study of the Aged (LEILA 75+). *Int. Psychogeriatr.* 23 1592–1601. 10.1017/S104161021100144X 21813037

[B17] Ehreke L. Luppa M. König H.-H. Riedel-Heller S. G. (2010). Is the clock drawing test a screening tool for the diagnosis of mild cognitive impairment? A systematic review. *Int. Psychogeriatr.* 22 56–63. 10.1017/S1041610209990676 19691908

[B18] Ehreke L. Luppa M. Luck T. Wiese B. Weyerer S. Eifflaender-Gorfer S. (2009). Is the clock drawing test appropriate for screening for mild cognitive impairment? – Results of the German study on ageing, cognition and dementia in primary care patients (AgeCoDe) for the AgeCoDe group. *Dement. Geriatr. Cogn. Disord.* 28 365–372. 10.1159/000253484 19887799

[B19] Elias M. F. Dore G. A. Goodell A. L. Davey A. Zilioli M. K. C. Brennan S. (2011). Normative data for elderly adults: the maine-syracuse study. *Exp. Aging Res.* 37 142–178. 10.1080/0361073X.2011.554511 21424955

[B20] Elliott R. Baker S. C. Rogers R. D. O’Leary D. A. Paykel E. S. Frith C. D. (1997). Prefrontal dysfunction in depressed patients performing a complex planning task: a study using positron emission tomography. *Psychol. Med.* 27 931–942. 10.1017/S0033291797005187 9234470

[B21] Fjell A. M. Walhovd K. B. (2010). Structural brain changes in aging: courses, causes and cognitive consequences. *Rev. Neurosci.* 21 187–221. 10.1515/REVNEURO.2010.21.3.187 20879692

[B22] Fransson P. Marrelec G. (2008). The precuneus/posterior cingulate cortex plays a pivotal role in the default mode network: evidence from a partial correlation network analysis. *Neuroimage* 42 1178–1184. 10.1016/J.NEUROIMAGE.2008.05.059 18598773

[B23] Freedman M. Leach L. Kaplan E. Winocur G. Shulman K. I. Delis D. C. (1994). *Clock Drawing: A Neuropsychological Analysis*. Oxford: Oxford University Press, 10.1017/CBO9781107415324.004

[B24] Funahashi S. (2001). Neuronal mechanisms of executive control by the prefrontal cortex. *Neurosci. Res.* 39 147–165. 10.1016/S0168-0102(00)00224-8 11223461

[B25] Garrett D. D. Lindenberger U. Hoge R. D. Gauthier C. J. (2017). Age differences in brain signal variability are robust to multiple vascular controls. *Sci. Rep.* 7:10149. 10.1038/s41598-017-09752-7 28860455 PMC5579254

[B26] Grabner R. H. Neubauer A. C. Stern E. (2006). Superior performance and neural efficiency: the impact of intelligence and expertise. *Brain Res. Bull.* 69 422–439. 10.1016/j.brainresbull.2006.02.009 16624674

[B27] Grady C. L. Ma Maisog J. Horwitz B. Ungerleider L. G. Mentis M. J. Salerno J. A. (1994). Age-related changes in cortical blood flow activation during visual processing of faces and location largay for the behavioral testing; Dr. Margaret Daube-Witherspoon for keeping the tomograph and automatic blood sampler working. *J. Neurosci.* 14 1450–1462. 10.1523/JNEUROSCI.14-03-01450.1994 8126548 PMC6577560

[B28] Greicius M. D. Krasnow B. Reiss A. L. Menon V. (2003). Functional connectivity in the resting brain: a network analysis of the default mode hypothesis. *Proc. Natl. Acad. Sci. U.S.A.* 100 253–258. 10.1073/pnas.0135058100 12506194 PMC140943

[B29] Greicius M. D. Supekar K. Menon V. Dougherty R. F. (2009). Resting-state functional connectivity reflects structural connectivity in the default mode network. *Cereb. Cortex* 19 72–78. 10.1093/cercor/bhn059 18403396 PMC2605172

[B30] Harada C. N. Natelson Love M. C. Triebel K. L. (2013). Normal cognitive aging. *Clin. Geriatr. Med.* 29 737–752. 10.1016/j.cger.2013.07.002 24094294 PMC4015335

[B31] Heinik J. Reider-Groswasser I. I. Solomesh I. Segev Y. Bleich A. (2000). Clock drawing test: correlation with linear measurements of CT studies in demented patients. *Int. J. Geriatr. Psychiatry* 15 1130–1137. 10.1002/1099-1166(200012)15:12<1130::AID-GPS259>3.0.CO;2-N 11180470

[B32] Hubbard E. J. Santini V. Blankevoort C. G. Volkers K. M. Barrup M. S. Byerly L. (2008). Clock drawing performance in cognitively normal elderly. *Arch. Clin. Neuropsychol.* 23 295–327. 10.1016/j.acn.2007.12.003 18243644 PMC2752157

[B33] Ino T. Asada T. Ito J. Kimura T. Fukuyama H. (2003). Parieto-frontal networks for clock drawing revealed with fMRI. *Neurosci. Res.* 45 71–77. 10.1016/S0168-0102(02)00194-3 12507726

[B34] Karimpoor M. Tam F. Strother S. C. Fischer C. E. Schweizer T. A. Graham S. J. (2015). A computerized tablet with visual feedback of hand position for functional magnetic resonance imaging. *Front. Hum. Neurosci.* 9:150. 10.3389/fnhum.2015.00150 25859201 PMC4373274

[B35] Kirby M. Denihan A. Bruce I. Coakley D. Lawlor B. A. (2001). The clock drawing test in primary care: sensitivity in dementia detection and specificity against normal and depressed elderly. *Int. J. Geriatr. Psychiatry* 16 935–940. 10.1002/gps.445 11607936

[B36] Kozora E. Cullum C. M. (1994). Qualitative features of clock drawings in normal aging and Alzheimer’s disease. *Assessment* 1 179–187. 10.1177/1073191194001002008 9465148

[B37] Lee D. Y. Seo E. H. Choo I. H. Kim S. G. Lee J. S. Lee D. S. (2008). Neural correlates of the clock drawing test performance in Alzheimer’s disease: a FDG-PET study. *Dement. Geriatr. Cogn. Disord.* 26 306–313. 10.1159/000161055 18841015

[B38] Leiner H. C. Leiner A. L. Dow R. S. (1993). Cognitive and language functions of the human cerebellum. *Trends Neurosci.* 16 444–447. 10.1016/0166-2236(93)90072-T 7507614

[B39] Mainland B. J. Shulman K. I. (2017). “Clock drawing test,” in *Cognitive Screening Instruments*, ed. Andrew L. (Cham: Springer International Publishing), 67–108.

[B40] Mason M. F. Norton M. I. Van Horn J. D. Wegner D. M. Grafton S. T. Macrae C. N. (2007). Wandering minds: the default network and stimulus-independent thought. *Science* 315 393–395. 10.1126/science.1131295 17234951 PMC1821121

[B41] Matsuoka T. Narumoto J. Okamura A. Taniguchi S. Kato Y. Shibata K. (2013). Neural correlates of the components of the clock drawing test. *Int. Psychogeriatr.* 258 1317–1323. 10.1017/S1041610213000690 23676356

[B42] Matsuoka T. Narumoto J. Shibata K. Okamura A. Nakamura K. Nakamae T. (2010). Neural correlates of performance on the different scoring systems of the clock drawing test. *Neurosci. Lett.* 487 421–425. 10.1016/j.neulet.2010.10.069 21055445

[B43] Mazziotta J. C. Toga A. W. Evans A. Fox P. Lancaster J. (1995). A probabilistic atlas of the human brain: theory and rationale for its development. The International Consortium for Brain Mapping (ICBM). *Neuroimage* 2 89–101. 10.1006/nimg.1995.1012 9343592

[B44] Mega M. S. Cummings J. L. (1994). Frontal-subcortical circuits and neuropsychiatric disorders. *J. Neuropsychiatry Clin. Neurosci.* 6 358–370. 10.1176/jnp.6.4.358 7841807

[B45] Menon V. Uddin L. Q. (2010). Saliency, switching, attention and control: a network model of insula function. *Brain Struct. Funct.* 214 655–667. 10.1007/s00429-010-0262-0 20512370 PMC2899886

[B46] Miller I. N. Himali J. J. Beiser A. S. Murabito J. M. Seshadri S. Wolf P. A. (2015). Normative data for the cognitively intact oldest-old: the framingham heart study. *Exp. Aging Res.* 41 386–409. 10.1080/0361073X.2015.1053755 26214098 PMC5621515

[B47] Ministry of Transportation Ontario (2018). *Ontario’s Age 80 and Above Licence Renewal Program*. Available at: http://www.mto.gov.on.ca/english/driver/pdfs/ges-practice-test-english.pdf [accessed March 5, 2018].

[B48] Nasreddine Z. S. Phillips N. A. Bedirian V. Charbonneau S. Whitehead V. Collin I. (2005). The montreal cognitive assessment, MoCA: a brief screening tool for mild cognitive impairment. *J. Am. Geriatr. Soc.* 53 695–699. 10.1111/j.1532-5415.2005.53221.x 15817019

[B49] Nishiwaki Y. Breeze E. Smeeth L. Bulpitt C. J. Peters R. Fletcher A. E. (2004). Validity of the clock-drawing test as a screening tool for cognitive impairment in the elderly. *Am. J. Epidemiol.* 160 797–807. 10.1093/aje/kwh288 15466502

[B50] Ogawa S. Lee T. M. Kay A. R. Tank D. W. (1990). Brain magnetic resonance imaging with contrast dependent on blood oxygenation. *Proc. Natl. Acad. Sci. U.S.A.* 87 9868–9872. 10.1073/PNAS.87.24.9868 2124706 PMC55275

[B51] Oldfield R. C. (1971). The assessment and analysis of handedness: the edinburgh inventory. *Neuropsychologia* 9 97–113. 10.1016/0028-3932(71)90067-4 5146491

[B52] Ott A. Breteler M. M. B. Van Harskamp F. Stijnen T. Hofman A. (1998). Incidence and risk of dementia: the rotterdam study. *Am. J. Epidemiol.* 147 574–580. 10.1093/oxfordjournals.aje.a009489 9521184

[B53] Paganini-Hill A. Clark L. J. Henderson V. W. Birge S. J. (2001). Clock drawing: analysis in a retirement community. *JAGS* 49 941–947. 10.1046/j.1532-5415.2001.49185.x 11527486

[B54] Park D. C. Reuter-Lorenz P. (2009). The adaptive brain: aging and neurocognitive scaffolding. *Annu. Rev. Psychol.* 60 173–196. 10.1146/annurev.psych.59.103006.093656 19035823 PMC3359129

[B55] Paula J. J. Miranda D. M. Moraes E. N. Malloy-Diniz L. F. (2013). Mapping the clockworks: what does the Clock Drawing Test assess in normal and pathological aging? *Arq. Neuropsiquiatr.* 71 763–768. 10.1590/0004-282X20130118 24212511

[B56] Picard N. Strick P. L. (2003). Activation of the Supplementary Motor Area (SMA) during performance of visually guided movements. *Cereb. Cortex* 13 977–986. 10.1093/cercor/13.9.977 12902397

[B57] Pinto E. Peters R. (2009). Literature review of the clock drawing test as a tool for cognitive screening. *Dement. Geriatr. Cogn. Disord.* 27 201–213. 10.1159/000203344 19225234

[B58] Porro C. A. Francescato M. P. Cettolo V. Diamond M. E. Baraldi P. Zuiani C. (1996). Primary motor and sensory cortex activation during motor performance and motor imagery: a functional magnetic resonance imaging study. *J. Neurosci.* 16 7688–7698. 10.1523/JNEUROSCI.16-23-07688.1996 8922425 PMC6579073

[B59] Rao S. M. Binder J. R. Bandettini P. A. Hammeke T. A. Yetkin F. Z. Jesmanowicz A. (1993). Functional magnetic resonance imaging of complex human movements. *Neurology* 43 2311–2318. 10.1212/WNL.43.11.2311 8232948

[B60] Raz N. Lindenberger U. Rodrigue K. M. Kennedy K. M. Head D. Williamson A. (2005). Regional brain changes in aging healthy adults: general trends, individual differences and modifiers. *Cereb. Cortex* 15 1676–1689. 10.1093/cercor/bhi044 15703252

[B61] Resnick S. M. Pham D. L. Kraut M. A. Zonderman A. B. Davatzikos C. (2003). Longitudinal magnetic resonance imaging studies of older adults: a shrinking brain. *J. Neurosci.* 23 3295–3301. 10.1523/JNEUROSCI.23-08-03295.2003 12716936 PMC6742337

[B62] Richardson H. E. Glass J. N. (2002). A comparison of scoring protocols on the clock drawing test in relation to ease of use, diagnostic group, and correlations with mini-mental state examination. *J. Am. Geriatr. Soc.* 50 169–173. 10.1046/j.1532-5415.2002.50024.x 12028263

[B63] Roberts A. C. Robbins T. W. Weiskrantz L. (1998). *The Prefrontal Cortex: Executive and Cognitive Functions*. New York, NY: Oxford University Press, 10.1093/acprof:oso/9780198524410.001.0001

[B64] Rouleau I. Salmon D. P. Butters N. Kennedy C. McGuire K. (1992). Quantitative and qualitative analyses of clock drawings in Alzheimer’s and Huntington’s disease. *Brain Cogn.* 18 70–87. 10.1016/0278-2626(92)90112-Y 1543577

[B65] Royall D. R. Cordes J. A. Polk M. (1998). CLOX: an executive clock drawing task. *J. Neurol. Neurosurg. Psychiatry* 64 588–594. 10.1136/JNNP.64.5.588 9598672 PMC2170069

[B66] Royall D. R. Mulroy A. R. Chiodo L. K. Polk M. J. (1999). Clock drawing is sensitive to executive control: a comparison of six methods. *J. Gerontol. Psychol. Sci.* 54 328–333. 10.1093/geronb/54B.5.P328 10542825

[B67] Rypma B. Berger J. S. Genova H. M. Rebbechi D. D’Esposito M. (2005). Dissociating age-related changes in cognitive strategy and neural efficiency using event-related fMRI. *Cortex* 41 582–594. 10.1016/S0010-9452(08)70198-9 16042034

[B68] Samton J. B. Ferrando S. J. Sanelli P. Karimi S. Raiteri V. Barnhill J. W. (2005). The clock drawing test: diagnostic, functional, and neuroimaging correlates in older medically ill adults. *J. Neuropsychiatry Clin. Neurosci.* 17 533–540. 10.1176/jnp.17.4.533 16387994

[B69] Shibasaki H. Sadato N. Lyshkow H. Yonekura Y. Honda M. Nagamine T. (1993). Both primary motor cortex and supplementary motor area play an important role in complex finger movement. *Brain* 116 1387–1398. 10.1093/brain/116.6.1387 8293277

[B70] Shoyama M. Nishioka T. Okumura M. Kose A. Tsuji T. Ukai S. (2011). Brain activity during the clock-drawing test: multichannel near-infrared spectroscopy study. *Appl. Neuropsychol.* 18 243–251. 10.1080/09084282.2011.595450 22074062

[B71] Shulman K. I. (2000). Clock-drawing: is it the ideal cognitive screening test? *Int. J. Geriatr. Psychiatry* 15 548–561. 10.1002/1099-1166(200006)15:6<548::AID-GPS242>3.0.CO;2-U 10861923

[B72] Siciliano M. Santangelo G. D’Iorio A. Basile G. Piscopo F. Grossi D. (2016). Rouleau version of the clock drawing test: age- and education-adjusted normative data from a wide Italian sample. *Clin. Neuropsychol.* 30 1501–1516. 10.1080/13854046.2016.1241893 27702066

[B73] Smith S. M. Jenkinson M. Woolrich M. W. Beckmann C. F. Behrens T. E. J. Johansen-Berg H. (2004). Advances in functional and structural MR image analysis and implementation as FSL. *Neuroimage* 23 S208–S219. 10.1016/j.neuroimage.2004.07.051 15501092

[B74] Spenciere B. Alves H. Charchat-Fichman H. (2017). Scoring systems for the clock drawing test: a historical review. *Dement. Neuropsychol.* 11 6–14. 10.1590/1980-57642016dn11-010003 29213488 PMC5619209

[B75] Stevens F. L. Hurley R. A. Taber K. H. (2011). Anterior cingulate cortex: unique role in cognition and emotion. *J. Neuropsychiatry Clin. Neurosci.* 23 121–125. 10.1176/jnp.23.2.jnp121 21677237

[B76] Strother S. C. Anderson J. Hansen L. K. Kjems U. Kustra R. Sidtis J. (2002). The quantitative evaluation of functional neuroimaging experiments: the NPAIRS data analysis framework. *Neuroimage* 15 747–771. 10.1006/nimg.2001.1034 11906218

[B77] Tam F. Churchill N. W. Strother S. C. Graham S. J. (2012). A new tablet for writing and drawing during functional MRI. *Hum. Brain Mapp.* 33 1750–1751. 10.1002/hbm.21375 PMC6870006 20336688

[B78] Thomann P. A. Toro P. Dos Santos V. Essig M. Schröder J. (2008). Clock drawing performance and brain morphology in mild cognitive impairment and Alzheimer’s disease. *Brain Cogn.* 67 88–93. 10.1016/j.bandc.2007.11.008 18215449

[B79] Tranel D. Rudrauf D. Vianna E. P. M. Damasio H. (2008). Does the clock drawing test have focal neuroanatomical correlates? *Neuropsychology* 22 553–562. 10.1037/0894-4105.22.5.553 18763875 PMC2834527

[B80] Turcotte V. Gagnon M.-E. Joubert S. Rouleau I. Gagnon J.-F. Escudier F. (2018). Normative data for the Clock Drawing Test for French-Quebec mid- and older aged healthy adults. *Clin. Neuropsychol.* 32 1–11. 10.1080/13854046.2018.1473495 29742962

[B81] Uddin L. Q. Nomi J. S. Hébert-Seropian B. Ghaziri J. Boucher O. (2017). Structure and function of the human insula. *J. Clin. Neurophysiol.* 34 300–306. 10.1097/WNP.0000000000000377 28644199 PMC6032992

[B82] Ueda H. Kitabayashi Y. Narumoto J. Nakamura K. Kita H. Kishikawa Y. (2002). Relationship between clock drawing test performance and regional cerebral blood flow in Alzheimer’s disease: a single photon emission computed tomography study. *Psychiatry Clin. Neurosci.* 56 25–29. 10.1046/j.1440-1819.2002.00940.x 11929568

[B83] van der Flier W. M. Scheltens P. (2005). Epidemiology and risk factors of dementia. *J. Neurol. Neurosurg. Psychiatry* 76(Suppl. 5), v2–v7. 10.1136/jnnp.2005.082867 16291918 PMC1765715

[B84] Velayudhan L. Ryu S.-H. Raczek M. Philpot M. Lindesay J. Critchfield M. (2018). Review of brief cognitive tests for patients with suspected dementia. *Int. Psychogeriatr.* 268 1247–1262. 10.1017/S1041610214000416 24685119 PMC4071993

[B85] Watson Y. I. Arfken C. L. Birge S. J. (1993). Clock completion: an objective screening test for dementia. *J. Am. Geriatr. Soc.* 41 1235–1240. 10.1111/j.1532-5415.1993.tb07308.x 8227899

[B86] White T. P. Joseph V. Francis S. T. Liddle P. F. (2010). Aberrant salience network (bilateral insula and anterior cingulate cortex) connectivity during information processing in schizophrenia. *Schizophr. Res.* 123 105–115. 10.1016/j.schres.2010.07.020 20724114

[B87] Yamamoto S. Mogi N. Umegaki H. Suzuki Y. Ando F. Shimokata H. (2004). The clock drawing test as a valid screening method for mild cognitive impairment. *Dement. Geriatr. Cogn. Disord.* 18 172–179. 10.1159/000079198 15211073

[B88] Yousry T. A. Schmid U. D. Alkadhi H. Schmidt D. Peraud A. Buettner A. (1997). Localization of the motor hand area to a knob on the precentral gyrus. A new landmark. *Brain* 120(Pt 1), 141–157. 10.1093/brain/120.1.141 9055804

[B89] Yu C. Zhou Y. Liu Y. Jiang T. Dong H. Zhang Y. (2011). Functional segregation of the human cingulate cortex is confirmed by functional connectivity based neuroanatomical parcellation. *Neuroimage* 54 2571–2581. 10.1016/j.neuroimage.2010.11.018 21073967

